# Vital Signs: Binge Drinking Among Women and High School Girls — United States, 2011

**Published:** 2013-01-11

**Authors:** Dafna Kanny, Yong Liu, Robert D. Brewer, Paul I. Eke, Shanna N. Cox, Nancy E. Cheal, Yvonne Green

**Affiliations:** Div of Population Health; Div of Reproductive Health, National Center for Chronic Disease Prevention and Health Promotion; Div of Birth Defects and Developmental Disabilities, National Center for Birth Defects and Developmental Disabilities; Office of Women’s Health, CDC

## Abstract

**Background:**

Excessive alcohol use accounted for an estimated average of 23,000 deaths and 633,000 years of potential life lost (YPLL) among women and girls in the United States each year during 2001–2005. Binge drinking accounted for more than half of those deaths and YPLL. Binge drinking also is a risk factor for many health and social problems among women and girls, including unintended and alcohol-exposed pregnancy, sexually transmitted diseases, and breast cancer.

**Methods:**

To describe the prevalence, frequency, and intensity of binge drinking (four or more drinks on an occasion in the last 30 days) among U.S. women aged ≥18 years, CDC analyzed data from the 2011 Behavioral Risk Factor Surveillance System. Data were also analyzed from the 2011 national Youth Risk Behavior Survey on the prevalence of current alcohol use (one or more drinks during the past 30 days) and binge drinking (five or more drinks in a row during the past 30 days) among U.S. high school girls in grades 9–12.

**Results:**

Among adult women, the prevalence of binge drinking was 12.5%, and among those who binge drank, the frequency of binge drinking was 3.2 episodes per month and the intensity was 5.7 drinks on occasion. Binge drinking was most prevalent among women aged 18–24 years (24.2%) and 25–34 years (19.9%), and among those from households with annual incomes of ≥$75,000 (16.0%). Among those who binge drank, women aged 18–24 years had the highest frequency (3.6 episodes) and intensity (6.4 drinks) of binge drinking. Among high school girls, the prevalence of current alcohol use was 37.9%, the prevalence of binge drinking was 19.8%, and the prevalence of binge drinking among girls who reported current alcohol use was 54.6%.

**Conclusions:**

Binge drinking is reported by one in eight U.S. adult women and one in five high school girls. Women who binge drink tend to do so frequently and with high intensity. Most high school girls who reported current alcohol use also reported binge drinking.

**Implications for Public Health Practice:**

More widespread implementation of evidence-based interventions, such as those recommended by the Guide to Community Preventive Services and the U.S. Preventive Services Task Force, would be expected to reduce the frequency and intensity, and ultimately the prevalence of binge drinking among women and girls, and the harms related to it.

## Introduction

Excessive alcohol use* among women and girls accounted for an estimated average of 23,000 deaths† and 633,00 years of potential life lost (YPLL)§ in the United States each year during 2001–2005. Binge drinking was responsible for more than half of those deaths and YPLL (1). Binge drinking is a risk factor for many health and social problems that affect women, including unintentional injuries, violence, liver disease, hypertension, heart disease, stroke, breast and other cancers, reduced cognitive function, and alcohol dependence (2). Binge drinking also can affect women’s reproductive health by increasing the risk for acquiring human immunodeficiency virus and other sexually transmitted infections, unintended pregnancy, miscarriage, and low birth weight (3). A woman who binge drinks might unintentionally expose a developing fetus to high blood alcohol concentrations, increasing the risk for sudden infant death syndrome, fetal alcohol spectrum disorder, and attention-deficit/hyperactivity disorder (3). At the state level, binge drinking by women correlates strongly with binge drinking by high school girls (4).

Reducing the prevalence of binge drinking among adults and youths¶ is a leading health indicator in *Healthy People 2020* (5). To assess measures of binge drinking nationwide among women and girls, CDC analyzed data from the 2011 Behavioral Risk Factor Surveillance System (BRFSS) to determine the prevalence, frequency, and intensity of binge drinking among adult women, and data from the 2011 national Youth Risk Behavior Survey (YRBS) to determine measures of current alcohol use and binge drinking among high school girls.

## Methods

### BRFSS

BRFSS is an annual, state-based, random-digit–dialed telephone survey of noninstitutionalized, civilian, U.S. adults aged ≥18 years that collects information on many leading health conditions and health risk behaviors, including binge drinking. In 2011, all 50 states and the District of Columbia (DC) conducted the BRFSS by landline and cellular telephones. The median proportion of all BRFSS interviews completed by cellular telephones was approximately 11%. In 2011, the median survey response rate was 49.7%, ranging from 33.8% to 64.1%.** BRFSS data were weighted to adjust for several demographic variables (e.g., education levels, marital status, home ownership, and telephone source). A total of 278,243 women respondents were included in the analysis. A more detailed description of BRFSS methods has been published (6).

For women, binge drinking was defined as consuming four or more alcoholic drinks per occasion during the past 30 days. Among women who binge drank, binge drinking frequency was defined as the total number of episodes of binge drinking during the past 30 days. Binge drinking intensity was defined as the average largest number of drinks consumed during the past 30 days by respondents who reported one or more episodes of binge drinking. Respondents who refused to answer, had a missing answer, or who answered “don’t know/not sure” were excluded from the analyses involving those variables.

### YRBS

The biennial national YRBS, a component of CDC’s Youth Risk Behavior Surveillance System, measures the prevalence of health risk behaviors among U.S. high school students. The 2011 national YRBS obtained cross-sectional data representative of public- and private-school students in grades 9–12 in all 50 states and DC. Students completed an anonymous, self-administered questionnaire that included questions about alcohol use. Students from 158 schools completed 15,503 questionnaires. The school response rate was 81%, the student response rate was 87%, and the overall response rate was 71%. After quality control measures were taken, data from 15,425 students were available for analysis, of which data from 7,536 student girls were included in the analysis. Data were weighted to adjust for school and student nonresponse and oversampling of black and Hispanic students. A more detailed description of YRBS methods has been published (7).

Current alcohol use was defined as having had at least one drink of alcohol on at least 1 day during the 30 days before the survey. Binge drinking was defined for girls and boys as having had five or more drinks of alcohol in a row (i.e., within a couple of hours) on at least 1 day during the 30 days before the survey. T-tests were used to test for significant (p<0.05) differences between subgroups. Respondents who did not respond to one or both questions were excluded from the analysis.

## Results

### BRFSS

In 2011, the overall prevalence of binge drinking among women aged ≥18 years was 12.5% (Table 1). Among women who binge drank, the frequency of binge drinking was 3.2 episodes per month and the intensity was 5.7 drinks on occasion. Binge drinking was most prevalent among women aged 18–24 years (24.2%) and 25–34 years (19.9%), and then gradually decreased with increasing age. The highest frequency (3.6 episodes) and intensity (6.4 drinks) of binge drinking was reported by women aged 18–24 years. The prevalence of binge drinking was highest among non-Hispanic white women (13.3%), but the frequency and intensity of binge drinking was similar across racial and ethnic groups. Women who did not graduate from high school had the lowest prevalence of binge drinking (8.5%), but those who binge drank had the highest frequency (4.2 episodes) and intensity (6.2 drinks) relative to women with higher educational levels. Binge drinking prevalence increased with household income, and was highest among women with annual household incomes of $75,000 or more (16.0%).

### YRBS

In 2011, the prevalence of current alcohol use and of binge drinking among high school girls in grades 9–12 was 37.9% and 19.8%, respectively (Table 2). Hispanic (22.4%) and non-Hispanic white (21.7%) high school girls had a higher prevalence of binge drinking than non-Hispanic black girls (10.3%). Binge drinking prevalence among high school girls increased with grade, and was twice as high among 12th grade girls (27.0%) as among 9th grade girls (13.0%).

The prevalence of binge drinking among high school girls who reported current alcohol use was 54.6% (Table 2). Non-Hispanic white (57.8%) and Hispanic (55.4%) high school girls who reported current alcohol use had a higher prevalence of binge drinking than non-Hispanic black (35.0%) high school girls who reported current alcohol use. The prevalence of binge drinking among high school girls who reported current alcohol use increased with grade, from 45.2% among girls in grade 9 to 61.7% among girls in grade 12.

## Conclusions and Comment

The results in this report indicate that in 2011, binge drinking was common among U.S. adult women, and women who binge drank tended to do so frequently (average of three times per month) and intensively (average of six drinks on occasion), placing themselves and others at a greater risk for alcohol-attributable harms (1,2). The prevalence of binge drinking was similar among high school girls (especially in grades 11 and 12), women aged 18–24 years, and women aged 25–34 years. Binge drinking was most prevalent among women living in households with annual incomes of $75,000 or more.

At the state level, alcohol consumption by high school girls is strongly correlated with alcohol consumption by adult women (4). This probably reflects the influence of adult drinking behavior on youths, including the fact that youths often obtain alcohol from adults (8) and that youths often aspire to behave like young adults. The drinking behavior of youths and adults also is affected by the price and availability of alcoholic beverages (9) and religious and cultural factors (10). Additionally, binge drinking, unlike other leading risk behaviors, has not been subjected to intense prevention efforts (11). Underage girls are overexposed to alcohol marketing relative to women to an even greater extent than underage boys are overexposed to alcohol marketing relative to men (12), thereby increasing the risk that girls will initiate alcohol consumption and consume more alcohol when they drink (13). New alcoholic beverages also have been developed and marketed (e.g., flavored malt beverages) that are known to appeal to underage girls (14).

Although binge drinking is more prevalent among men (15), women who binge drink are at high risk for alcohol-attributable harms, in part because they differ from men in their physiologic response to alcohol consumption. Women tend to reach higher blood alcohol levels than men at the same consumption level, even after taking into account differences in body size, food consumption, and other factors (16). In addition, binge drinking increases the risk for unintended pregnancy, and women with unintended pregnancies tend to have delayed pregnancy recognition (3), increasing the risk for alcohol-exposed pregnancy and adverse reproductive health outcomes, such as fetal alcohol spectrum disorder, among women who binge drink, and further emphasizing the need to prevent binge drinking in women.

The findings in this report are subject to at least five limitations. First, BRFSS and YRBS data are self-reported. Among adults, alcohol consumption generally, and excessive drinking in particular, are underreported in surveys because of recall bias and social desirability bias (17). A recent study using BRFSS data found that self-reports identify only 22%–32% of presumed alcohol consumption in states, based on alcohol sales (18). Second, BRFSS does not collect information from persons living in institutional settings (e.g., on college campuses and military bases); therefore, BRFSS data might not be representative of these populations. Third, the BRFSS median response rate in 2011 was 49.7%. Fourth, the YRBS data apply only to youths who attend school, and thus are not representative of all persons in this age group. Nationwide, in 2009, of persons aged 16–17 years, approximately 4% were not enrolled in a high school program and had not completed high school.†† Finally, the YRBS definition of binge drinking (five or more drinks in a row), is not gender-specific, and studies among women have shown that reducing the threshold for defining binge drinking from five drinks to four drinks increases the relative prevalence of binge drinking by more than one third (19).

The Guide to Community Preventive Services has recommended several population-level, evidence-based strategies to effectively reduce binge drinking and related harms. These include 1) limiting alcohol outlet density, 2) holding alcohol retailers liable for harms related to the sale of alcoholic beverages to minors and intoxicated patrons (dram shop liability), 3) maintaining existing limits on the days and hours when alcohol is sold, 4) measures increasing the price of alcohol, 5) avoiding further privatization of alcohol sales in states with government-operated or contracted liquor stores, 6) electronic screening and brief interventions in the clinical setting, and 7) maintaining and enforcing age 21 years as the minimum age for legal drinking (20). The U.S. Preventive Services Task Force also recommends screening and behavioral counseling interventions for alcohol misuse, including binge drinking, among adults (21). The findings of this study also support the need to monitor binge drinking routinely among women and girls (11,15) to characterize the public health impact of this behavior, and to evaluate the effect of evidence-based strategies to prevent it.

**Key Points**

- Binge drinking is responsible for more than half of the estimated 23,000 deaths and 633,000 years of potential life lost among women and girls because of excessive alcohol consumption in the United States.
- In 2011, more than 13.6 million (12.5%) U.S. adult women binge drank (prevalence) an average of three times a month (frequency), and consume on average six drinks on occasion (intensity).
- The prevalence and intensity of binge drinking was highest among women aged 18–24 years.
- Women with household incomes ≥$75,000 had the highest binge drinking prevalence.
- In 2011, more than one in three high school girls reported drinking and one in five reported binge drinking; most high school girls who drank reported binge drinking.
- More widespread implementation of evidence-based interventions, such as those recommended by the Guide to Community Preventive Service and by the U.S. Preventive Services Task Force, would reduce binge drinking in states, as well as the health and social harms related to it.
- Additional information is available at http://www.cdc.gov/vitalsigns.

## Figures and Tables

**TABLE 1:** Binge drinking* prevalence, frequency, and intensity, by sociodemographic characteristics among women — Behavioral Risk Factor Surveillance System, United States,† 2011

	Prevalence			Frequency§			Intensity¶		
			
Characteristic	No.	Weighted %	(95% CI)	No.	No. of episodes	(95% CI)	No.	No. of drinks	(95% CI)
**Total**	**278,243**	**12.5**	**(12.2–12.8)**	**24,681**	**3.2**	**(3.1–3.3)**	**23,352**	**5.7**	**(5.6–5.8)**
**Age groups (yrs)**									
18–24	10,378	24.2	(22.7–25.6)	2,535	3.6	(3.3–3.9)	2,381	6.4	(6.1–6.6)
25–34	26,042	19.9	(19.0–20.7)	5,023	3.0	(2.8–3.2)	4,786	6.0	(5.9–6.2)
35–44	35,290	14.5	(13.8–15.1)	5,049	3.0	(2.8–3.2)	4,808	5.5	(5.4–5.6)
45–64	112,529	9.5	(9.2–9.9)	9,957	3.3	(3.2–3.5)	9,427	5.1	(5.0–5.2)
≥65	94,004	2.5	(2.3–2.7)	2,117	3.4	(3.0–3.8)	1,950	4.2	(4.1–4.4)
**Race/Ethnicity**									
White, non-Hispanic	219,519	13.3	(13.0–13.7)	19,969	3.3	(3.2–3.4)	19,033	5.7	(5.6–5.8)
Black, non-Hispanic	24,521	10.1	(9.3–10.9)	1,670	3.2	(2.9–3.5)	1,524	5.2	(5.0–5.4)
Hispanic	17,089	11.0	(10.1–11.9)	1,545	2.8	(2.5–3.1)	1,414	5.8	(5.5–6.1)
Other, non-Hispanic**	14,625	10.9	(9.7–12.2)	1,369	3.4	(2.8–4.0)	1,272	6.0	(5.6–6.4)
**Education Level**									
Less than high school diploma	24,036	8.5	(7.7–9.3)	1,335	4.2	(3.6–4.8)	1,186	6.2	(5.8–6.6)
High school diploma	82,247	10.9	(10.4–11.4)	6,136	3.4	(3.2–3.7)	5,720	5.9	(5.7–6.1)
Some college	78,925	14.3	(13.7–14.9)	7,636	3.3	(3.2–3.5)	7,237	5.7	(5.6–5.8)
College graduate	92,528	14.1	(13.6–14.6)	9,552	2.7	(2.6–2.8)	9,189	5.3	(5.2–5.4)
**Income**									
<$25,000	78,723	11.4	(10.8–11.9)	5,533	3.4	(3.2–3.6)	5,155	6.0	(5.9–6.2)
$25,000–$49,999	63,946	12.0	(11.5–12.6)	5,546	3.3	(3.1–3.6)	5,261	5.8	(5.7–6.0)
$50,000–$74,999	35,840	13.0	(12.2–13.7)	3,690	2.9	(2.7–3.1)	3,567	5.4	(5.3–5.6)
≥$75,000	57,364	16.0	(15.4–16.7)	7,547	3.0	(2.8–3.2)	7,261	5.4	(5.2–5.5)

**Abbreviation:** CI = confidence interval.

* For women, binge drinking was defined in the BRFSS as consuming four or more alcoholic drinks per occasion during the past 30 days.

† Respondents were from 50 states and the District of Columbia.

§ Binge drinkers only; average number of binge-drinking episodes per month.

¶ Average largest number of drinks consumed by binge drinkers on any occasion in the past month.

** Other, non-Hispanic includes Asian, Native Hawaiian or other Pacific Islander, American Indian or Alaskan Native, other race, and multiracial.

**TABLE 2:** Prevalence of current alcohol use and binge drinking* by race/ethnicity and grade among high school girls — National Youth Risk Behavior Survey, United States, 2011

	Current alcohol use (N = 7,032)		Binge drinking (N = 7,536)		Binge drinking among students reporting current alcohol use (N = 2,745)	
			
Characteristic	%	(95% CI)	%	(95% CI)	%	(95% CI)
**Total**	**37.9**	**(36.1–39.8)**	**19.8**	**(18.6–21.1)**	**54.6**	**(52.6–56.5)**
**Race/Ethnicity**						
White, non-Hispanic	38.8	(36.1–41.6)	21.7	(20.0–23.5)	57.8	(55.0–60.4)
Black, non-Hispanic	31.6	(28.0–35.3)	10.3	(8.3–12.6)	35.0	(28.6–42.1)
Hispanic	42.4	(39.4–45.5)	22.4	(20.5–24.5)	55.4	(52.4–58.4)
Other, non-Hispanic†	31.7	(27.1–36.6)	16.7	(13.7–20.1)	55.4	(46.8–63.8)
**Grade**						
9	30.3	(27.2–33.6)	13.0	(10.9–15.3)	45.2	(40.5–50.0)
10	37.1	(33.9–40.3)	17.8	(15.9–19.9)	50.4	(46.0–54.8)
11	40.1	(36.9–43.3)	22.6	(19.9–25.4)	58.4	(53.9–62.8)
12	45.4	(41.6–49.4)	27.0	(23.8–30.6)	61.7	(57.2–66.0)

**Abbreviation:** CI = confidence interval.

* Defined in the YRBS for girls and boys as having had five or more drinks of alcohol in a row (i.e., within a couple of hours) on at least 1 day during the 30 days before the survey.

† Other, non-Hispanic includes Asian, Native Hawaiian or other Pacific Islander, American Indian or Alaskan Native, and multiracial.

## Footnotes

* Excessive alcohol use includes binge drinking (defined by CDC as consuming four or more drinks per occasion for women or five or more drinks per occasion for men), heavy drinking (defined as consuming more than one drink per day on average for women or more than two drinks per day on average for men), any alcohol consumption by pregnant women, and any alcohol consumption by youths aged <21 years.

† Alcohol-attributable deaths for 2001–2005 were estimated using the Alcohol-Related Disease Impact (ARDI) application. Additional information is available at http://apps.nccd.cdc.gov/dach_ardi/default/default.aspx.

§ YPLL for 2001–2005 were estimated using the ARDI application using death and life expectancy data from the National Vital Statistics System.

¶ Objective SA-14.3 (adults) and SA-14.4 (youth). Objective MICH-11.2 (pregnant women).

** Response rates for BRFSS are calculated using standards set by the American Association of Public Opinion Research (AAPOR) response rate formula no. 4, available at http://www.aapor.org/standard_definitions2.htm. The response rate is the number of respondents who completed the survey as a proportion of all eligible and likely eligible persons. Additional information is available at http://www.cdc.gov/brfss/technical_infodata/quality.htm.

†† Information is available at http://nces.ed.gov/pubs2012/2012006.pdf.
