# Erratum

**Published:** 2013-01-18

## Vol. 62, No. 1

In the report, “Vital Signs: Binge Drinking Among Women Excessive alcohol use^*^ among women and girls accounted for an estimated average of 23,000 deaths^†^ and **633,000** years of potential life lost (YPLL)^§^ in the United States each year during 2001–2005.” and High School Girls—United States, 2011,” one of the numbers in the first sentence of the Introduction was incorrectly stated. The sentence should read as follows: “

